# Evaluation of functional outcomes following rectus diastasis repair—an up-to-date literature review

**DOI:** 10.1007/s10029-021-02462-0

**Published:** 2021-07-24

**Authors:** A. Olsson, O. Kiwanuka, G. Sandblom, O. Stackelberg

**Affiliations:** 1 Department of Clinical Science and Education, Södersjukhuset, and Department of Surgery, Södersjukhuset, Karolinska Institute, Sjukhusbacken 10, 11883 Stockholm, Sweden; 2 Institute of Environmental Medicine, Unit of Cardiovascular and Nutritional Epidemiology, Karolinska Institute, Stockholm, Sweden

**Keywords:** Abdominal rectus muscle diastasis, Diastasis recti, Abdominoplasty, Functional outcome, Urinary incontinence, Quality of life, Surgery

## Abstract

**Introduction:**

Over the last decade rectus diastasis has gained attention as a condition that may benefit from surgery. Numerous surgical techniques have been presented but scientifically proper studies reporting functional outcome are few and evidence is incomplete. The aim of this up-to-date review is to analyse the outcomes of rectus diastasis repair in recently published papers, focusing on functional changes following surgery.

**Method:**

A comprehensive search in PubMed and Web of Science was performed. Suitable papers were selected using titles and abstracts with terms suggesting surgical treatment of rectus diastasis. All abstracts were scrutinised, and irrelevant studies excluded in four stages. Reports providing original data, including outcome assessment following surgery, were included.

**Result:**

Ten papers with a total of 780 patients were found to fulfil the search criteria. Study design, surgical procedure, follow-up time, functional outcome and assessment instruments were compiled. All included studies reported improvements in a variety of functional aspects regardless of surgical method. The outcomes assessed include core stability, back pain, abdominal pain, posture, urinary incontinence, abdominal muscle strength and quality of life.

**Conclusion:**

The results of this review show that surgical repair of rectus diastasis is a safe and effective treatment that improves functional disability. However, the absence of standardized instruments for assessing outcome makes it impossible to compare studies. Since indications for surgery are relative and related to core function, valid instruments for assessing indication and outcome are needed to ensure benefit of the procedure.

## Introduction

The condition *rectus diastasis* is characterised by a vertical abnormal separation of the rectus abdominis muscles [1]. The widened and weakened linea alba in rectus diastasis often presents as a bulging or sagging of the abdominal midline. The condition should not be mistaken for a hernia, although the risk for concomitant midline hernias seems to be more common with a present rectus diastasis [2–4]. The main risk factors for developing rectus diastasis are pregnancy and obesity, due to distention and increased intraabdominal pressure [5]. During pregnancy mechanical and hormonal changes contribute to the extension of the entire abdominal wall, including a widening of the linea alba, causing the rectus diastasis. The abdominal wall usually recovers after pregnancy, but in some women an abdominal laxity persists and causes abdominal core instability. The prevalence for persisting rectus diastasis is reported to approximately 30–40% [5, 6]. Rectus diastasis in postpartum women has gained increasing attention during the last decade. The condition is associated with functional disabilities, e.g., core instability and back pain, as well as abdominal deformations, such as bulging, all causing different degrees of functional and aesthetic disturbances. Multiple studies have reported the association between rectus diastasis and functional disabilities [7–9].

Treatment options include conservative management with core stability training, surgical reconstruction of the widened midline, or a combination of both [10]. Several training methods have been described [11] that seem to increase core strength and stability but fail to significantly reduce the diastasis [12, 13]. Positive results after surgical reconstruction of rectus diastasis has been reported in multiple studies [14]. A repair of the rectus diastasis is a common component in abdominoplasty, primarily performed for cosmetic reasons [15]. As functional disabilities associated with rectus diastasis has gained acknowledgement, the need for surgical reconstruction with functional indications has increased. Considering this wider indication, surgical repair is not only relevant for plastic surgeons but for general surgeons as well.

Numerous different surgical techniques for rectus diastasis repair have been described during the last decade, open as well as laparoscopic, with mesh reinforcement as well as suture repair [16, 17]. The various surgical methods reported in the literature have been evaluated regarding recurrences and postoperative complications [14]. There is, however, a paucity of patient-reported outcomes regarding functional improvements, such as back pain, core stability, abdominal muscle strength, urinary incontinence as well as quality of life. These are potential debilitating symptoms affecting many post-partum women. The purpose of this review study was to summarize reported outcomes after surgical reconstruction of rectus diastasis, in terms of physical function and quality of life.

## Methods

The literature review was designed and reported according to the PRISMA 2020 guidelines [18]. A literature review of published clinical studies reporting functional outcomes and postoperative complications after surgical repair of rectus diastasis with the indication core instability symptoms was conducted.

Inclusion criteria for selecting studies were: articles in English language; participants ≥ 18 years of age; evaluation of physical function prior to surgery and at follow-up; surgical method including a full repair of the rectus diastasis. Exclusion criteria were: systematic reviews; narrative reviews; conference abstracts; case reports; studies without adequate evaluation of outcome; articles in other language than English.

A systematic data collection was performed during March and April 2021. Searches were conducted in PubMed. The search strategy was developed in PubMed: (Abdominal Rectus Diastasis´[Mesh]) OR (Rectus Diastasis´[Mesh]) OR (Diastasis recti Abdominis´[Mesh]) OR (Diastasis Rectus Abdominis Muscles´[Mesh] OR (Abdominal Rectus Diastasis´[Text Word]) OR (Rectus Diastasis´[Text Word]) OR (Diastasis recti Abdominis´[Text Word]) OR (Diastasis Rectus Abdominis Muscles´[Text Word]). The final search was performed 25 April 2021.

Study screening procedure was performed by retrieving titles and abstracts. All abstracts were evaluated, and irrelevant studies were excluded in four steps. All relevant abstracts were reviewed and studies fulfilling inclusion criteria were included. Supplemental inclusion of relevant studies from reference lists was performed.

Extraction of relevant data variables was conducted regarding: author, study design, number of patients, surgical approach, diastasis reconstruction technique, follow-up time, recurrence, postoperative complications, such as hematoma, seroma, surgical site infections, information regarding preoperative and postoperative physical function (back pain, abdominal muscle strength, core stability, posture, abdominal pain, pulmonary function, intra-abdominal pressure, urinary incontinence), and information regarding preoperative and postoperative mental health and quality of life.

## Results

**Fig. 1:**
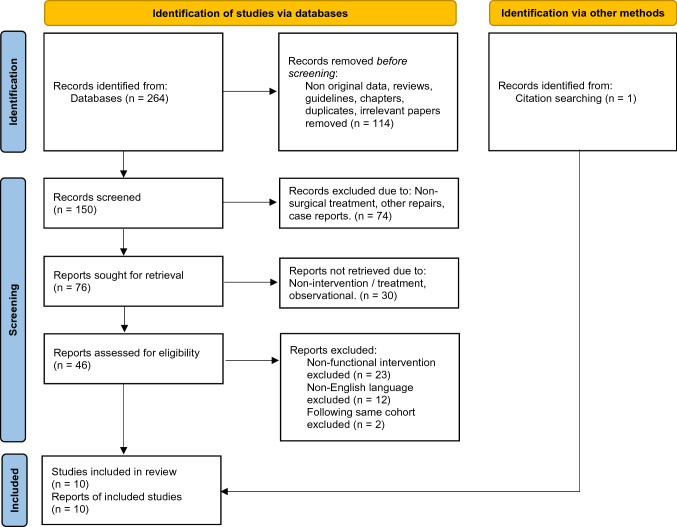
Flow chart of inclusion and exclusion of retrieved studies

The review of current literature identified 264 studies, of which 10 studies met the inclusion criteria [19–28], Fig. 1. The reports fulfilling these criteria included two randomized controlled studies (RCTs) [21, 28] and eight prospective cohort studies [19, 20, 22–27].

### Characteristics of participants

The total study population consisted of 780 individuals. The demographics of participants and indications for surgery across the studies are shown in Table 1.

**Table 1:** Characteristics of participants and indications for surgery

Study	Refs.	(*n*)	Age	Sex	BMI (kg/m^2^)	Indication for surgery
Bellido et al. (Spain)	[19]	21	Mean 37.6 (range 24–50)	18 women 3 men	Mean: 27.4 (range 22–35)	Midline hernias combined with rectus diastasis
Carrara et al. (Italy)	[20]	110	Mean 43.1 (range 27–81)	102 women 8 men	Mean: 21.5 (range 17.5–30.5)	Midline hernias combined with rectus diastasis
Emanuelsson et al. (Sweden)	[21]	A: 27 B: 29 C: 30	Median: A:39.6 (range 29–61) B: 42 (range 27–62) C: 44.2 (range 29–67)	87 women 2 men	Median: A: 23 (range 18–37) B: 23 (range 18–30) C: 22.8 (range 18–30)	Rectus diastasis combined with functional disabilities
Manetti et al. (Italy)	[22]	74	Mean 46.3 (SD 11.3)	65 women 9 men	Mean: 24.3 (SD 4.1)	Rectus diastasis combined with associated symptoms
Olsson et al. (Sweden)	[23]	60	Mean 38.8 (range 20.5–53)	60 women	Mean: 22.6 (range 17.2–36)	Training resistant symptomatic rectus diastasis
Pereira et al. (Chile)	[24]	10	Mean 43.5 (range 34–60)	10 women	Mean: 27.2 (range23.2–33.3)	Patients planned for abdominoplasty
Rodrigues et al. (Brazil)	[25]	18	Mean 31 (range 21–50)	18 women	Mean: 23.9 (range 20–28)	Patients planned for abdominoplasty
Taylor et al. (Australia)	[26]	214	Mean 42.1 (range 24–65)	214 women	Mean 26.3 (range 19–39)	Postpartum women planned for abdominplasty
Temel et al. (Turkey)	[27]	40	Mean 43.8 (range 33–48)	40 women	Mean not reported (range 18.5– > 40)	Rectus diastasis combined with abdominal muscle weakness, and back and lumbar pain
Wilhelmsson et al. (Sweden)	[28]	A: 101 B: 75	Mean A: 47.5 (range 25–72) B: 49.1 (range 25–72)	A: 61 women 5 men B: 53 women 6 men	A: 27.1 (SD 2.4) B: 26.2 (SD 1.9)	Patients with skin excess planned for abdominoplasty

A summary of assessment instruments, follow-up time and functional outcomes as well as surgical methods, recurrencies and common postoperative complications are shown in Table 2.

**Table 2:** Characteristics of included studies, surgical technique, follow-up times, outcome, and assessment methods

Study	Refs.	Study design	(*n*)	Surgical method	FU	Recurrency	Postoperative complications	Functional outcome	Instrument
Bellido et al. (Spain)	[19]	Prospective cohort study	21	Laparoscopic suture repair + mesh	1 year	0	Seroma 23% (*n* = 5/21), Subcutaneous emphysema 9.5% (*n* = 2/21)	Back pain	VAS
Carrara et al. (Italy)	[20]	Prospective cohort study	110	Laparoscopic staple repair + mesh	2 years	0	Haematoma 4.5% (*n* = 5/102) SSI 3.6% (*n* = 4/102) Seroma 0.9% (*n* = 1/102) Internal hernia 0.9% (*n* = 1/102)	QoL, low back pain, urinary incontinence	EuraHSQol, ODI, ISI
Emanuelsson et al. (Sweden)	[21]	RCT	A: 29 B: 28	A: Open suture repair B: Open suture + mesh	1 year	1 (group A)	Seromas 8.8% (*n* = 5/57)	Abdominal muscle strength, abdominal pain, QoL	Biodex System 4, VHPQ, SF-36
Manetti et al. (Italy)	[22]	Prospective cohort study	74	Laparoscopic suture repair + mesh	6 months	2	Haematoma 2.7% (*n* = 2/74)	Back pain, abdominal swelling, shortness of breath, urinary incontinence	PROM
Olsson et al. (Sweden)	[23]	Prospective cohort study	60	Open suture repair	1 year	0	Haematoma 6.7% (*n* = 4/60) Seroma 6.7% (*n* = 4/60) SSI 3.3% (*n* = 2/60) Spontaneous pneumothorax 1.7% (*n* = 1/60)	Self-rated physical function, Abdominal/back/trunk muscle endurance, urinary incontinence, QoL	ATFP, DRI, SF-36, UDI7, IIQ6
Pereira et al. (Chile)	[24]	Prospective cohort study	10	Open suture repair	Peri-operative	not reported	not reported	Pulmonary function, intra-abdominal pressure	P-Comp, transvesical pressure
Rodrigues et al. (Brazil)	[25]	Prospective cohort study	18	Open suture repair	15 days	not reported	not reported	Ventilatory function, intra-abdominal pressure	Spirometri, transvesical pressure
Taylor et al. (Australia)	[26]	Multicenter prospective study	214	Open suture repair	6 months	not reported	not reported	Back pain, urinary incontinence	Oswestry Disability Index, ICIQ
Temel et al. (Turkey)	[27]	Prospective cohort study	40	Open suture repair	6 months	not reported	Seroma 5% (*n* = 2/40) SSI 5% (*n* = 2/40)	Posture, Back pain, QoL, depression	Radiography, VAS, BDI, NPH
Wilhelmsson et al. (Sweden)	[28]	RCT	A: 101 B: 75	A: Open suture repair B: Non-repair	1 year	not reported	not reported	Trunk muscle endurance, lung function, self-rated physical function	Physio, PEF, DRI

### Characteristics of included studies

Bellido et al [19], from the Abdominal Surgery, Minimal Invasive Surgery Departments in Huelva, Sevilla and Granada in Spain, showed in a prospective cohort study from 2015, a significant improvement in back pain 20 months after surgery. Twenty-one patients with midline hernias and associated rectus diastasis were included during 2011–2012. The surgical technique was a full endoscopic subcutaneous approach with suture approximation of the diastasis with non-resorbable suture plicating the anterior rectus sheets, combined with onlay mesh reinforcement. Back pain was rated on a Visual Analogic Scale preoperatively and 12 months after surgery.

Carrara et al [20], from the General Surgery Unit at Ospedale Santa Chiara in Trento, Italy, presented a prospective observational study on 110 consecutive patients with midline hernias combined with rectus diastasis in 2020. Surgical procedure was performed with laparoscopic approach with a stapler closure of the posterior rectus sheets combined with a retromuscular mesh reinforcement, the THT technique [29]. Back pain was assessed with the Oswestry Disability Index questionnaire, Quality of Life was evaluated with EuraHSQol, urinary incontinence was evaluated with the Incontinence Severity Index.

Emanuelsson et al [21], from the Reconstructive Plastic Surgery and the Centre for Surgical Gastroenterology at the Karolinska University Hospital in Stockholm Sweden, presented a randomized controlled trial in 2016. Fifty-seven patients with diagnosed rectus diastasis combined with a history of functional disabilities, such as back pain, abdominal pain or weakness, were randomized to either open repair with resorbable suture plication, or to resorbable suture plication combined with retromuscular mesh reinforcement. There was a non-surgical control group consisting of 32 patients undergoing physiotherapy. At the 1-year follow-up abdominal pain was evaluated with the Ventral Hernia Pain Questionnaire, abdominal wall muscle strength was evaluated with the Biodex System-4, Quality of Life was evaluated with the SF-36 questionnaire, patient perceived muscle strength was evaluated with a Visual Analog Scale. A long term follow-up has been presented by Swedenhammar et al [30], in 2020. At the 5-year follow-up, there were still no recurrencies, no difference between the two groups regarding Quality of Life (SF-36), or self-reported muscle strength (VAS). The preoperatively reported pain had decreased significantly at the 5-year follow-up.

Manetti et al [22], from the Department of General Surgery at St. Giovanni Addolorata Hospital in Rome Italy, reported a series of laparoscopic repairs in a prospective cohort study in 2021. Seventy-four patients with diagnosed rectus diastasis (> 2 cm) combined with associated symptoms, were included and underwent laparoscopic surgery with a stapler closure of the anterior rectus sheets combined with a retromuscular mesh. Functional symptoms, including urinary incontinence, lower back pain, shortness of breath and abdominal swelling, were evaluated with a questionnaire preoperatively and 6 months postoperatively.

Olsson et al [23], from Södersjukhuset in Stockholm, Sweden, presented a cohort study 2019, where 60 consecutive patients with diagnosed rectus diastasis (> 3 cm) combined with training resistant abdominal core instability symptoms, were operated with open repair with resorbable sutures. Functional outcomes were assessed prior to and 1 year after surgery, with a self-report questionnaire, the Disability Rating Index and seven functional tests supervised and monitored by a physiotherapist. The functional tests were compiled in the Abdominal Trunk Function Protocol (ATFP), Quality of Life was evaluated with the SF-36 questionnaire. Urinary incontinence was evaluated with the Urogenital Distress Inventory (UDI-6) and the Incontinence Impact Questionnaire (IIQ-7),

Pereira et al [24], from the Plastic Surgery Department at Hospital del Trabajador in Santiago, Chile presented a prospective cohort study in 2016, exploring the perioperative changes of intraabdominal pressure and pulmonary function in ten patients planned for abdominoplasty. Intraabdominal pressure was measured with a modified Kron´s trans-bladder technique and pulmonary function was measured with pulmonary compliance (P-Comp). Assessments were performed intraoperatively before and after plication of the diastasis.

Rodrigues et al [25], from the Plastic Surgery Department at the Federal University in Sao Paolo, Brazil, presented a prospective cohort study in 2013, assessing ventilatory function and intra-abdominal pressure postoperatively. Eighteen patients diagnosed with rectus diastasis and abdominal deformity III or B according to the Nahas classification, [31] underwent surgery with plication of the diastasis as well as plication of the external oblique. Ventilatory function was evaluated with spirometry prior to surgery as well as 2, 7 and 15 days after surgery. Intra-abdominal pressure was evaluated by measuring the intravesical pressure.

Taylor et al [26], presented a multicenter prospective cohort study in 2017, where nine private plastic surgery clinics in Australia participated. In total 214 postpartum women planned for abdominoplasty were included to the study. Back pain and urinary incontinence were evaluated prior to and 6 months after abdominoplasty with rectus diastasis repair included. Back pain was assessed with the Oswestry Disability Index and urinary incontinence was assessed with the International Consultation on Incontinence Questionnaire.

Temel et al [27], from the Division of Plastic Surgery at the Mustafa Kemal University in Hatay, Turkey, presented a prospective cohort study in 2016, where 40 women planned for abdominoplasty due to skin excess, were studied regarding postural and psychological effects of abdominoplasty including vertical rectus plication, 6 months after surgery. Posture was evaluated with bidirectional radiography of the thoracic and the lumbar regions, defining lumbar lordosis, thoracic kyphosis and the lumbosacral angle, 6 months after surgery. Back and lumbar pain was assessed with a Visual Analog Scale. Depression severity was assessed with the Beck Depression Inventory. Quality of Life was evaluated with the Nottingham Health Profile.

Wilhelmsson et al [28], presented a randomized clinical trial from the Department of Surgery and the Department of Plastic Surgery, Sahlgrenska Hospital in Gothenburg Sweden 2017. In total 125 patients planned for abdominoplasty due to excess skin were randomized to abdominoplasty with or without rectus abdominis plication. Trunk muscle endurance was assessed with an abdominal static endurance test and an extensor static endurance test. Self-reported physical function was assessed with the Disability Rating Index questionnaire. Lung function was assessed with a peak expiratory test. All functional tests were performed prior to and 1 year after surgery.

### Surgical technique

Seven studies analysed open repair [21, 23–28], of which one RCT compared suture repair (absorbable) with and without mesh reinforcement [21] and one RCT compared abdominoplasty with suture repair (non-absorbable), with abdominoplasty without diastasis repair [28]. Five of the prospective cohort studies on open repair used absorbable sutures in one case [23] and non-absorbable sutures in three cases [24, 25, 27], while one study did not report data on suture material [26]. Three studies analysed laparoscopic repairs, one used stapling closure of the anterior rectus sheets combined with sublay mesh reinforcement [20]; one used stapling closure of the posterior rectus sheets combined with sublay mesh reinforcement [22]; and the third used suture repair of the anterior rectus sheets combined with onlay mesh reinforcement [19], Table 2.

### Functional outcome

#### Posture

One study examined posture [27] evaluated as vertebral-column angles. Significant changes were seen in mean thoracic kyphosis angle (*p* < 0.001), mean lumbar lordosis angle (*p* < 0.001), mean lumbosacral angle (*p* < 0.001), indicating an improved posture, 6 months after surgery.

#### Abdominal pain

Abdominal pain was evaluated in one RCT [21], showing a significant decrease of abdominal pain (pain last week, score > 1), in both groups at 1-year follow-up, using the VHPQ [21].

#### Abdominal muscle strength

Abdominal muscle strength was assessed in three studies [21, 23, 28]. Emanuelsson et al. examined abdominal muscle strength with using the Biodex System-4 in a RCT, and reported significant improvements in isometric strength, flexion strength and extension strength 1 year after surgery, regardless of repair technique. [21] One study examined abdominal muscle endurance using a standardized physical test monitored by a physiotherapist (the ATFP), reported an improvement from 49 to 66 s 1 year after surgery (not statistically significant) [23]. Wilhelmsson et al. examined abdominal muscle endurance. They showed a slight improvement which was not statistically significant [28], and there were no differences seen between the two groups examined.

#### Abdominal trunk muscle endurance

One study on trunk muscle endurance using functional tests monitored by a physiotherapist (ATFP) showed significant improved trunk endurances (trunk stability, side plank), with a mean change from 40 to 56 s (*p* < 0.001) 1 year after surgery [23].

#### Back muscle strength

A study on back muscle strength, using a standard physical test, showed a decrease in endurance in the non-plication group (*p* = 0.02) that was not seen in the plication group [28].

#### Lung function

Four studies reported changes in lung function perioperatively [24], early postoperatively [25], 6 months after surgery [22] and at 1-year follow-up. [28] Pereira et al. reported a significant decrease of perioperative pulmonary compliance (P-Comp) measured before and after plication of the rectus diastasis, pre-plication score 38.98, and post-plication score 36.54 (*p* = 0.0076) [24]. Rodrigues et al. examined ventilatory function using spirometry preoperatively and postoperatively until day 15 after surgery. There was an initial significant reduction in spirometry values postoperatively, but these recovered close to preoperative values by day 15 [25]. Manetti et al. reported a statistically significant decrease of self-reported respiratory symptoms, mainly “shortness of breath”, from 28% (*n* = 21) to 4% (*n* = 3) [22]. Wilhelmsson et al. assessed peak expiratory flows, showing no change at 1-year follow-up regardless of repair technique: non-plication group (*p* = 0.42), plication group (*p* = 0.41) [28].

### Patient-reported outcome

#### Back pain

Five studies reported a significant decrease in back pain [19, 20, 22, 26, 27]. Bellido et al. assessed back pain using a Visual Analogic Scale, preoperatively and 12 months after surgery. Follow-up showed a significant decrease of self-reported back pain, from 4.3 to 2.2 (*p* < 0.001) [19]. Carrara et al. reported a significant decrease in back pain, 6 months after surgery, using the Oswestry Disability Index questionnaire, from 11.5 to 2.6 (*p* < 0.0017) [20] Manetti et al. reported a statistically significant decrease of occurrence of lower back pain from 54% (*n* = 40/74) to 5% (*n* = 7/74), using a questionnaire completed by 77% (*n* = 57/74) participants. Taylor et al. reported a decrease of back pain with values changing from 10.9 to 1.58 (*p* < 0.001) 6 months after surgery, using the Oswestry Disability Index questionnaire [26]. BMI and presence of an umbilical hernia were predictors for preoperative back pain. Temel et al. evaluated back pain 6 months after surgery, with a Visual Analogic Scale (scale: 0–100) reporting a significant decrease from preoperative score 83.3 ± 10 to 17 ± 7.2 (*p* < 0.001) [27].

#### Physical function

Three studies were found to report changes in self-rated physical function [21, 23, 28]. One study reported a patient-reported improvement in functional disabilitiy in 98% (*n* = 59/60) [23]. One study reported patient perceived muscle strength evaluated at the 1-year follow-up, using a Visual Analog Scale, showing significant improvements similar in the two groups, compared to the training group (*p* < 0.001), while the third study reported a significant improvement in only one subscale (running) in the plication group (*p* = 0.04), but no significant change in the compiled questionnaire [28].

Urinary incontinence was evaluated in four studies [20, 22, 23, 26]. Carrara et al. reported a significant decrease of score in the Incontinence Severity Index questionnaire (from 3.6 to 0.7), 6 months after surgery, (*p* < 0.0001) [20]. Manetti et al. reported a statistically significant decreased occurrence of urinary incontinence from 42% (*n* = 31/74) preoperatively to 3% (*n* = 2/74) postoperatively [22]. Olsson et al. showed significant decreased scores in the questionnaires Urinary Distress Inventory (UDI-6), with median score change from 5 to 2 (*p* = 0.001), and Incontinence Impact Questionnaire (IIQ-7), with median score change from 2 to 0 (*p* = 0.002), at follow-up 1 year after surgery [23]. Taylor et al. reported a significant decreased score, examined with the International Consultation on Incontinence Questionnaire at follow-up 6 months after surgery. the change of mean score was 6.22 preoperatively to 1.60 postoperatively (*p* < 0.001) [26].

#### Abdominal swelling

One study evaluated abdominal swelling using a self-report questionnaire, showing a statistically significant decrease from 60 to 9 at the 6-month follow-up [22].

#### Quality of life

Four studies evaluated health-related quality of life [20, 21, 23, 27]. Carrara et al. evaluated QoL with the EuraHSQol showing a significant improvement at 6-month follow-up (*p* < 0.0001) [20]. One RCT evaluated QoL with the SF-36 questionnaire showing significant improvements (*p* < 0.001) in most subscales at the 1-year follow-up, and no differences between groups [21]. One cohort study evaluated QoL with the SF-36 and showed statistically significant improvements in all subscales at the 1-year follow-up [23]. Temel et al. evaluated QoL with the Nottingham Health Profile questionnaire, showing significant improvements in fatigue, pain and sleep at the 6-month follow-up [27].

#### Psychiatry

One study assessed depression using the BDI questionnaire [27]. At the 6-month follow-up the BDI scores showed that depression had decreased significantly (*p* < 0.001).

## Discussion

This literature review provides a comprehensive overview of current knowledge regarding patient-reported-, and functional outcomes after rectus diastasis repair. We can report that the outcome is assessed with various instruments, focusing on core stability, back pain, abdominal pain, posture, urinary incontinence, abdominal muscle strength and quality of life. Most studies showed improvement in patient-reported outcomes and the overall conclusion is that surgical repair provides improved physical function, decreased urinary incontinence and improved quality of life.

The reviewed studies cover different aspects of the most common functional symptoms associated with postpartum rectus diastasis. The clinical studies are accomplished by general surgeons as well as plastic surgeons. Furthermore, surgical techniques vary and include endo-laparoscopic methods as well as open methods. Surgical procedures are carried out in University hospitals, public hospitals and in private clinics. The studies were conducted in different regions worldwide, including Europe, South America, Asia and Australia. The diversity of the studies origins, designs and outcomes underline the summarized findings.

In most of the included studies significant improvements in measured functional outcomes were reported. Surgical repair provides an improved self-reported physical function, abdominal core muscle strength, posture, and quality of life, as well as decreased urinary incontinence and lower back pain. Surgical repair, regardless of method, seems to result in physical benefits.

Back pain is one of the most common functional disabilities associated with rectus diastasis [32, 33]. The mechanism has not yet been described in detail but the reported improvements in back pain, posture, and core stability, in several of the reviewed articles underlines the importance of maintaining the function of the abdominal cannister.

The change in pulmonary function and intra-abdominal pressure seems to be temporary and the initial postoperative reduction in pulmonary function and increase in intra-abdominal pressure were normalized quickly after surgery. The reported significant increased intra-abdominal pressure was associated with the use of an abdominal binder [24]. A normal physical function seems to adapt to the new postoperative condition and the functional changes were considered not clinically relevant in healthy individuals [25]. Caution should be taken in patients with concomitant pulmonary deficiencies.

Several studies reported a reduction of urinary incontinence. This finding indicates that a repair of the ventral abdominal wall possibly provides stability to all parts of the abdominal cannister, including the pelvic floor. An improved strength and stability of the ventral abdominal muscles after surgical repair seems to improve the function of the pelvic floor which is not reported after physiotherapy alone. This finding underlines that the abdominal cannister is an integrated system of collaborating muscle groups.

Quality of life was improved in all studies measuring the quality-of-life parameter. It is not possible to separate the impact of functional improvements from a possible improved aesthetic result following surgery on quality of life in these studies. On the other hand, the functional and the aesthetic aspect of this issue could be considered as two sides of the same coin. An adequate physical function, as well as the aesthetic perception, possibly affect the quality of life in substantial proportions.

The absence of standardized methods to assess the effectiveness and safety of the different therapies and surgical approaches, as well as the lack of control groups, make it hard to compare the outcomes. The lack of conformity and standardized instruments for outcome measures make it difficult to compare the results and impossible to draw definite conclusions.

In the reviewed studies, surgical repair of rectus diastasis has a low frequency of recurrency and postoperative complications, and a mild but temporarily negative impact on lung function and intra-abdominal pressure without clinical relevancy. This review study suggests that surgery is a safe and effective treatment that could be considered in patients with persisting disabling functional symptoms associated with rectus diastasis. It is not, however, possible to recommend any specific surgical method prior to other methods from this study.

### Limitations

Despite the intention to cover all relevant reports, this review study may not be fully complete. There are a limited number of studies included in this review and there is a potential risk of missing important reports. Some of the included reviewed studies may uphold a low evidence level. The outcomes are on the other hand pointing towards different aspects of improved functions following surgery which indicates a promising future for surgical management of rectus diastasis.

## Conclusion

In conclusion, the findings in this review study confirm the potential functional benefit of surgical repair in patients with persistent symptomatic rectus diastasis. There is a great need of standardized instruments for measuring the outcome after surgery for rectus diastasis. Even if the outcomes from the reviewed studies indicate mostly favourable outcomes, it is possible that the reported improvement in abdominal core function reflects a placebo effect of surgery in studies, where controls are lacking. Whereas no studies have shown that any surgical technique is superior to the others, further research is necessary, and to that end we must have valid instruments for assessing indication for and outcome of surgery.

## Data Availability

Not applicable.
